# Supplementary material for: Topographical analysis of arterial perforators in the face: A cadaveric study
Source: PLoS One. 2026 May 14;21(5):e0349023. doi: 10.1371/journal.pone.0349023 (PMC13175362; doi:10.1371/journal.pone.0349023)
Supplement: Attachment — Submitted filename: Response to Reviewers.docx [file pone.0349023.s002.docx]

**Response to Reviewers**

**Topographical Analysis of Arterial Perforators in the Face: A Cadaveric Study**

***PLOS ONE***

**April 16, 2026**

We sincerely thank the editor and reviewer for the careful review of our manuscript and for the constructive comments that helped us improve the quality and clarity of the manuscript. We have revised the manuscript accordingly, and our detailed responses are provided below.

**Comment 1. Relationship with clinically used facial flaps**

**Response 1.:**

Thank you for this valuable comment. In response, we have expanded the Discussion section to clearly describe the clinical relevance of our findings in relation to commonly used facial flaps. Specifically, we added a new paragraph (Lines 243–253) explaining how the perforator distribution map can be applied in clinical practice.

In this revision, we emphasized that the identified high-density perforator regions correspond to areas frequently used in flap design, and we described their implications from both donor and recipient perspectives. These additions aim to provide a more concrete explanation of how the anatomical findings can be translated into practical surgical applications.

**Comment 2. Clarity and concision of the Discussion**

**Response 2.:**

We appreciate the editor’s suggestion to improve the clarity and conciseness of the Discussion section. In response, we have carefully revised the section by removing repetitive descriptions of numerical results that were already presented in the Results section.

In addition, references related to clinically meaningful perforator diameter thresholds have been reorganized and presented in a more concise manner to improve readability and allow for easier comparison.

**Comment 3. Minor language and typographical corrections**

**Response 3.:**

Following the editor’s suggestion, the manuscript has undergone professional English language editing. We have corrected grammatical errors and typographical inconsistencies throughout the manuscript to improve overall clarity and readability.

We hope that these revisions have adequately addressed your comments and improved the manuscript. We sincerely appreciate your consideration.

**Prof. Seung-Ho HAN, M.D., Ph.D.**

**Department of Anatomy, Ewha Womans University College of Medicine**
